# Accelerometry Correlates in Body Composition, Physical Fitness, and Disease Symptom Burden: A Pilot Study in End-Stage Renal Disease

**DOI:** 10.3389/fphys.2021.737069

**Published:** 2021-12-07

**Authors:** Vedrana Sember, Špela Bogataj, Jose Carlos Ribeiro, Armin Paravlić, Maja Pajek, Jernej Pajek

**Affiliations:** ^1^Faculty of Sport, University of Ljubljana, Ljubljana, Slovenia; ^2^Department of Nephrology, University Medical Centre, Ljubljana, Slovenia; ^3^Faculty of Sports, Research Centre in Physical Activity Health and Leisure, University of Porto, Porto, Portugal; ^4^Science and Research Centre, Institute of Kinesiology Research, Koper, Slovenia

**Keywords:** physical activity, chronic kidney disease, dialysis, accelerometers, monitoring, body composition, physical fitness, disease burden

## Abstract

There is strong evidence that hemodialysis (HD) patients with a sedentary lifestyle have a higher risk of death compared to peers who engage in regular physical activity. Therefore, monitoring physical activity is of utmost importance. However, there is a lack of data on objectively measured physical activity behaviors in HD patients. Therefore, this study aimed to objectively measure physical activity in HD patients throughout the week, with particular attention to dialysis and non-dialysis days. We also examined how objectively measured physical activity correlated with physical fitness, body composition, and disease burden. Daily physical activity, body composition, serum parameters, comorbidity index, sit-to-stand, and hand-grip strength tests were measured in 14 HD patients. Daily physical activity was measured using the Actigraph GT9X accelerometer. The Dialysis Symptom Index questionnaire was also used. We found significant differences in anthropometric variables (weight, body mass index, overhydration, lean tissue index, and fat tissue index, all *p* < 0.05) and phase angle (*p* < 0.01) between HD patients reaching and patients not reaching physical activity guidelines for patients with chronic diseases. HD patients showed to be less active during dialysis days compared to non-dialysis days as indicated in sedentary time (–11.7%; *p* = 0.001), light (–47.3%; *p* = 0.003), moderate (–51.5%; *p* = 0.001), moderate to vigorous (–49.3%; *p* = 0.001), and vigorous (–34.3%; *p* = 0.067) physical activity. No significant correlations were found among serum parameters, symptom burden, and comorbidity burden, but a very large and positive correlation was found between phase angle and total moderate to vigorous physical activity (*p* < 0.01). Our findings support the need to implement physical activity on dialysis days in HD units to mitigate the effects of sedentary behavior. Prospective, long-term studies evaluating the use of accelerometers in HD patients and their effects on physical activity are needed.

## Introduction

Patients with chronic kidney disease (CKD) suffer from high morbidity and mortality associated with cardiovascular disease and poor quality of life (Foley et al., 1995; Codreanu et al., 2006). In addition, body weakness and sarcopenia limit their physical performance (Roshanravan et al., 2017). Physical inactivity is a contributing factor to the debility in patients with CKD (Johansen et al., 2000). Patients on hemodialysis (HD) with a sedentary lifestyle have a higher risk of death compared to peers who engage in regular physical activity (O’Hare et al., 2003). There is a lack of objective assessment of physical activity in populations with chronic diseases. The use of accelerometers to objectively measure physical activity in HD patients is increasing (Young et al., 2019). Compared to accelerometers, the International Physical Activity Questionnaire (IPAQ) underestimates light physical activity, which is the main form of physical activity in HD patients (da Costa Rosa et al., 2015). Several studies used accelerometers to measure physical activity in the HD population (Kim et al., 2014; Gomes et al., 2015; Kopple et al., 2015; Broers et al., 2017), where the main findings revealed significantly lower physical activity compared to matched sedentary renal disease-free controls, with the lowest physical activity on HD days (Kim et al., 2014; Gomes et al., 2015; Kopple et al., 2015). A study from Japan found no difference between physical activity on dialysis and non-dialysis days (Morishita et al., 2014), which contrasts with physical activity patterns in Western populations (Young et al., 2019), and highlights the need for more studies with objectively measured physical activity. Specifically, we need additional data on the extent of decline in habitual physical activity in dialysis patients, influence of dialysis procedures themselves, and characteristics of patient subgroups at greatest risk for inactivity.

The World Health Organization (WHO) recommendation for physical activity in chronic conditions includes at least 150–300 min of moderate-intensity aerobic physical activity per week (Bull et al., 2020). However, physical activity is low in HD patients, especially on HD days (Young et al., 2019), and we need to understand this phenomenon better. There are many causes of reduced physical activity in dialysis patients, and time spent undergoing HD treatment is certainly one of them (da Costa Rosa et al., 2015). HD patients are required to spend 4–5 h in a recumbent position three times per week during HD. Afterward, they report post-dialysis burnout, which may also be related to their reduced physical activity (Johansen et al., 2005).

Compared to healthy individuals, HD patients have reduced phase angle (Bellizzi et al., 2006). Phase angle is a linear method of measuring the relationship between electric and reactance signals determined by bioimpedance analysis and represents the arc tangent value of the ratio of reactance to electrical resistance (Kumar et al., 2012). It is known as a predictor of body cell mass, indicator of cell integrity, and is considered prognostic, nutritional, health, and functional indicator in various chronic disease populations (Grundmann et al., 2015; Ruiz-Margáin et al., 2015; Alves et al., 2016). Bioimpedance phase angle was also found to predict health-related quality of life, muscle function, hospitalizations, and mortality in HD patients (Beberashvili et al., 2014).

Information on physical activity correlates in patients with end-stage renal disease would be useful to identify factors responsible for low levels of physical activity and, possibly, poor adherence to structured exercise interventions. Also, it would help us identify patients with greatest benefit from structured exercise interventions and develop interventions to improve physical activity and health-related physical fitness in HD patients. However, there are only a limited number of studies on correlates of objectively measured physical activity in patients on dialysis treatment, and additional data are needed on the difference in physical activity between dialysis and non-dialysis days objectively measured by accelerometers.

In this pilot study, we objectively measured physical activity in HD patients during whole dialysis week, such as weekdays and weekends. We aimed to assess physical activity levels during dialysis and non-dialysis days, physical fitness, body composition, and disease symptom burden of HD patients. Specifically, we looked at the magnitude of physical activity differences in dialysis vs. non-dialysis days and compared subjective characteristics of patients not reaching and reaching physical activity recommendations (at least 30 min of MVPA/day, five times/week). We also examined how objectively measured physical activity correlates with physical fitness, body composition, and disease burden.

## Materials and Methods

### Subjects

The data for this study were acquired from the baseline data of a randomized controlled trial investigating medium cut-off dialysis and fiber supplementation to reduce residual uremic syndrome (registered at clinicaltrials.gov, NCT04247867). This analysis was performed as a cross-sectional study on trial’s baseline data examining the physical fitness, physical activity, disease burden, and biochemical parameters of chronic prevalent hemodialysis patients. They were eligible for inclusion in the study if aged at least 18 years old, could walk with or without additional support, and had voluntarily given informed written consent to be included in the study. Patients were not included if any of the following conditions were present: hospitalization or acute illness in the last weeks preceding study measurements, active malignant disease or chronic infection (e.g., tuberculosis and osteomyelitis), consequences of cerebrovascular accident (such as paresis or paralysis), heart failure of New York Heart Association stage 3 or 4 or symptomatic angina pectoris Canadian Cardiovascular Society stage 2, 3, or 4, chronic obstructive pulmonary disease stage 3 or 4, decompensated liver cirrhosis, symptomatic peripheral arterial obstructive disease, painful degenerative or inflammatory arthroplasty with current use of anti-inflammatory or analgesic therapy, or a currently symptomatic psychiatric condition. Altogether, 14 patients participated (*n* = 7 males).

**FIGURE 1:**
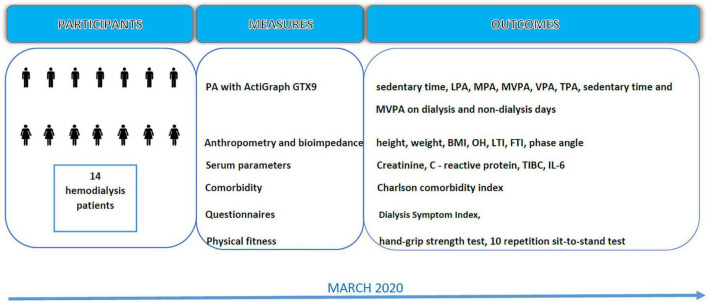
Data collection flowchart. LPA, light physical activity; MPA, moderate physical activity; MVPA, moderate to vigorous physical activity; TPA, total time physical activity; BMI, body mass index; OH, over hydration; LTI, lean tissue index; FTI, fat tissue index; TIBC, total iron binding capacity; IL-6, interleukin 6.

The data for this study were collected in March 2020 from the outpatient Slovenian dialysis unit at University Medical Centre Ljubljana (see Flowchart, Figure 1). All the participants were notified about the purpose and procedures of data collection, and their written consent was obtained prior to the measurements. The research protocol was reviewed and approved by the Slovenian National Medical Ethics Committee (ref. no. 0120-430/2019/12). This study was carried out following the Declaration of Helsinki guidelines for human research.

### Procedures

#### Physical Fitness, Body Composition, Disease Symptom Burden, and Biochemistry

We assessed the muscular strength of both hands with a handgrip dynamometer (Jamar Smart Digital Hand Dynamometer; Performance Health International Ltd.) and functional lower extremity strength by 10-repetition sit-to-stand test (10STS). A bioimpedance spectroscopy body composition monitor (BCM, Fresenius Medical Care, Bad Homburg, Germany) was used to measure lean tissue index, fat tissue index, extracellular overhydration, and phase angle. All the subjects were measured prior to midweek HD procedure. The conditions during the measurement were stable in terms of air temperature (23°C) and humidity (58%). All bioimpedance measurements were performed in accordance with the manufacturers’ user manuals. Disease symptom burden was assessed with the Dialysis Symptom Index questionnaire translated and validated for Slovene language (Weisbord et al., 2004; Heric et al., 2021). Venous serum samples were taken from the arterial dialysis line prior to start of the midweek HD procedure.

#### Physical Activity

We used the accelerometer Actigraph GT9X (Actigraph LLC, Pensacola, FL, United States) applied to patients for seven complete days, comprising of: 5 weekdays and 2 weekend-days. The patients wore an accelerometer placed on the right hip using an adjustable belt all the time and only removed it during water-based activities, such as personal hygiene and swimming. A trained team of researchers delivered the ActiGraph devices on a Monday or Wednesday morning prior to dialysis and collected the device the following Tuesday or Thursday after the dialysis. The functioning of the ActiGraphs was explained to the patients prior to the start of the monitoring period. In addition, short written guidelines for the proper use of the equipment were prepared and given to the patients.

We collected data at 50 Hz, and count signals were sampled in 1-sec epoch to achieve the most accurate assessment of their habitual physical activity. Physical activity data were logical and coincided with diary logs. Wear time was defined as from 7:00 to 21:30 (870 min) and valid day was when the awake wearing time (non-sleeping data) was >600 min (Choi et al., 2011). A total of 7 days of valid data were included in the analyses. Physical activity of all the patients was measured on the same week to minimize the effects of weather conditions on patients’ habitual physical activity. The main outcomes of reduced data were total physical activity (TPA), sedentary time, light physical activity (LPA), moderate physical activity (MPA), vigorous physical activity (VPA), and moderate to vigorous physical activity (MVPA). To determine the time spent in physical activity of different intensities, the following counts intervals (counts/min) were considered: 0–99 for sedentary time, 100–2,019 for LPA, 2,020–5,998 for MPA, and ≥5,999 for VPA (Troiano et al., 2008).

#### Statistical Analysis

Data were analyzed using the IBM SPSS Statistics 24.0 software for Windows (SPSS Inc., Chicago, IL, United States) and Microsoft Excel 2016 (Microsoft Corp., Dublin, Ireland). Physical activity data were processed with the Actilife software (version 6.3.14, standardized for accelerometer ActiGraph). For the purpose of presenting physical activity data, dialysis (3 days of the total week) and non-dialysis days (4 days of the total week) were combined. Moreover, physical activity data were also combined based on patients reaching physical activity recommendations (rMVPA group, *n* = 7) and patients not reaching physical activity recommendations (nrMVPA group, *n* = 7) (Bull et al., 2020). All the data are presented as mean ± SD. Descriptive statistics were used to summarize patient demographic characteristics and outcomes of interest. Normality of data distribution was confirmed by visual inspection and the Shapiro-Wilk test, while the homogeneity of variances was tested by Levene’s test for all dependent variables. Student’s *t*-test was performed to compare accelerometer data from dialysis days with those from non-dialysis days, and for HD patients who achieved MVPA recommendations compared to those who did not. In addition, the relationship between the two most representative physical activity domains (sedentary time and sum of moderate and vigorous activity time) and variables related to patient health status was examined using Pearson’s Correlation Coefficient. Statistical significance was set at the level of *p* < 0.05.

## Results

All the patients achieved accelerometer wear compliance of whole dialysis week. Thus, the mean days of wearing the accelerometer were 3 and 4 during dialysis and non-dialysis days, respectively. The mean accelerometer wearing time per day was 21.19 ± 2.8 h, with a significant difference (percentage difference [PD] = 17.1%; *p* = 0.001) between dialysis (1,171.2 ± 198 min/day) and non-dialysis days (1,390.2 ± 72.6 min/day).

The recruited patients had HD vintage of 9.5 ± 9.2 years (range between 1 and 31 years) and have been diagnosed with renal disease for 17.66 ± 11.74 years (range between 4 and 48 years). Their demographic and clinical data are given in Table 1. No significant differences in physical activity and other variables except for handgrip strength between male and female patients were found. There were no statistically significant differences between groups in duration of CKD (*p* = 5.03), vintage time (*p* = 0.984), and weekly dialysis time (*p* = 0.184). When we compared the subgroup of patients reaching and not reaching WHO recommendations for physical activity, we found significant differences in their body composition: weight (*p* = 0.022), BMI (*p* = 0.031), overhydration (*p* = 0.033), lean tissue index (*p* = 0.024), and phase angle (*p* = 0.002), and no differences in other health-related variables, such as comorbidity score, symptom burden, physical fitness, and biochemistry parameters. Also, patients not reaching physical activity recommendations were older and had a larger dialysis symptom index; however, this did not reach statistical significance in this sample size.

**TABLE 1:** Basic demographic characteristics of hemodialysis patients and comparisons between males and females and patients reaching and not reaching physical activity recommendations.

	All subjects	Males (*N* = 7)	Females (*N* = 7)	nrMVPA (*N* = 7)	rMVPA (*N* = 7)	RMD nrMVPA vs. rMVPA

**Parameters**	**Mean (SD)**	**Mean (SD)**	**Mean (SD)**	**Mean (SD)**	**Mean (SD)**	
**Anthropometric parameters**						
Age (years)	57.00 (10.38)	55.14 (9.42)	58.86 (11.70)	60.29 (11.88)	53.71 (8.20)	6.57
Height (cm)	168.36 (10.57)	169.86 (12.14)	166.86 (9.46)	166.71 (9.52)	170.00 (12.06)	−3.29
Weight (kg)	69.61 (13.42)	72.69 (16.97)	66.54 (8.96)	**61.80 (11.01)**	**77.43 (11.25)**	−15.63*
BMI (kg/m^2^)	24.57 (4.14)	25.26 (5.60)	23.89 (2.17)	**22.27 (3.56)**	**26.87 (3.48)**	−4.60*
Overhydration	1.59 (1.81)	1.04 (1.78)	2.13 (1.81)	**2.59 (1.79)**	**0.59 (1.26)**	2.00*
Lean tissue index	13.12 (2.39)	13.56 (3.29)	12.69 (1.06)	**11.74 (1.29)**	**14.50 (2.51)**	−2.76*
Fat tissue index	10.76 (4.08)	11.16 (5.52)	10.36 (2.30)	9.47 (3.24)	12.04 (4.66)	−2.57
Phase angle	4.94 (1.08)	5.14 (1.37)	4.74 (0.73)	**4.16 (0.71)**	**5.72 (0.78)**	−1.55**
**Disease and comorbidity burden**						
CKD duration (yrs.)	17.66 (11.74)	19.32 (14.85)	16.01 (8.47)	19.87 (14.83)	15.45 (8.21)	4.42
Dialysis vintage time (yrs)	9.54 (9.22)	10.04 (8.42)	9.05 (10.62)	9.60 (10.54)	9.49 (8.55)	0.11
Dialysis time (hrs/w)	13.68 (1.48)	13.71 (1.70)	13.64 (1.35)	13.14 (1.63)	14.21 (1.19)	−1.07
Charslon Score (points)	4.36 (2.21)	3.71 (2.14)	5.00 (2.24)	4.71 (2.06)	4.00 (2.45)	0.71
Dialysis symptom ind.	11.86 (11.55)	6.00 (1.73)	17.71 (14.36)	16.00 (14.71)	7.71 (5.74)	8.29
**Physical fitness**						
Sit-to-stand test*	19.50 (3.61)	18.31 (2.64)	20.68 (4.24)	19.92 (3.75)	19.08 (3.70)	0.84
Hand grip** (kg)	31.90 (9.60)	26.27 (3.78)*	37.53 (10.56)*	33.99 (10.99)	29.81 (8.30)	4.17
**Serum parameters**						
Creatinine (μmol/L)	735.86 (114.50)	691.14 (56.56)	780.57 (143.33)	769.57 (154.60)	702.14 (43.08)	67.43
High-sensitivity CRP (mg/L)	3.21 (4.40)	1.08 (0.65)	5.34 (5.56)	2.81 (2.54)	3.62 (5.92)	−0.80
Interleukin 6 (ng/L)	10.49 (7.62)	9.61 (6.96)	11.36 (8.69)	13.00 (9.55)	7.97 (4.44)	5.03
Hemoglobin (g/L)	121.29 (10.87)	123.00 (8.16)	119.57 (13.51)	121.86 (13.47)	120.71 (8.60)	1.14
Total iron-binding capacity (μmol/L)	41.86 (5.26)	41.49 (5.95)	42.24 (4.91)	42.33 (4.75)	41.40 (6.07)	0.93
Albumin (g/L)	40.93 (3.05)	41.71 (3.20)	40.14 (2.91)	39.71 (2.87)	42.14 (2.91)	−2.43
**Physical activity (min/day; the results shownin the table are the averaged sum of dialysis and non-dialysis days)**						
Sedentary time	1142.20 (205.11)	1083.40 (248.92)	1201.00 (145.33)	1232.46 (180.23)	1051.93 (199.14)	180.53
LPA	93.94 (70.31)	85.81 (54.28)	102.05 (87.25)	74.52 (56.39)	113.34 (81.57)	−38.82
MPA	30.81 (17.66)	34.40 (23.83)	27.23 (8.82)	**17.07 (9.55)**	**44.56 (11.99)**	−27.48**
MVPA	35.33 (18.94)	39.71 (25.80)	35.42 (8.20)	**20.49 (11.61)**	**50.17 (11.34)**	−29.68**
VPA	4.66 (3.52)	5.31 (4.49)	3.72 (2.30)	3.42 (2.38)	5.62 (4.29)	−2.2
Step counts	3883.80 (2063.35)	4272.45 (2602.62)	3495.14 (1448.57)	**2604.92 (914.79)**	**5162.67 (2138.07)**	−2557.76*

Ind., index; PA, physical activity; LPA, light physical activity; MPA, moderate PA; MVPA, moderate to vigorous physical activity; VPA, vigorous physical activity; CKD, chronic kidney disease; RMD, raw mean difference; nrMVPA, not reaching MVPA recommendations; rMVPA, reaching MVPA recommendations. Bold text is used to mark significant differences. *p value < 0.05; **p value < 0.001.

**FIGURE 2:**
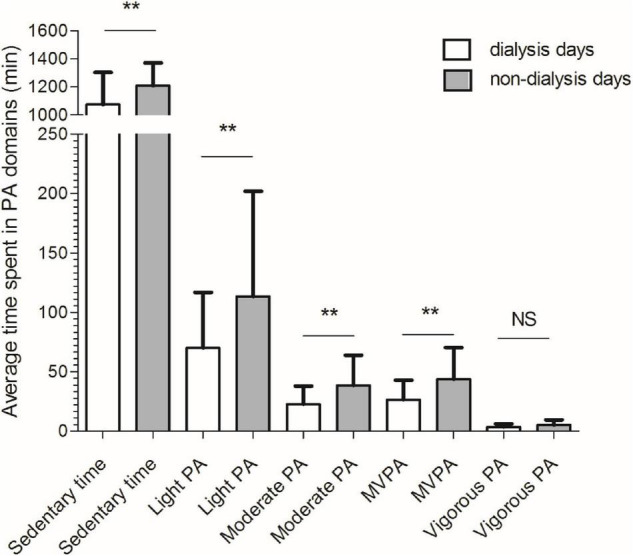
Comparisons between dialysis (white bar) and non-dialysis (gray bar) for time spent in different physical activity domains. PA, physical activity; **p < 0.01; NS, non-significant.

A significant difference was observed for time spent in all physical activity domains between dialysis and non-dialysis days, except for VPA (Figure 2). The HD patients were, in average, physically less active during dialysis days compared to non-dialysis days on following levels of physical activity: sedentary time (–11.7%; *p* = 0.001), light physical activity (–47.3%; *p* = 0.003), moderate physical activity (–51.5%; *p* = 0.001), moderate to vigorous physical activity (–49.3%; *p* = 0.001), and vigorous physical activity (–34.3%; *p* = 0.067).

**FIGURE 3:**
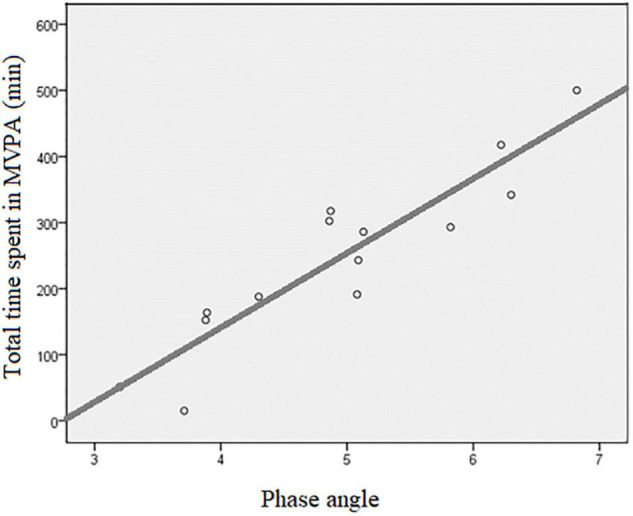
Scatter plot of the association between total time spent in MVPA and phase angle. MVPA, moderate to vigorous physical activity; r, Pearson’s product-moment correlation coefficient = 0.919, *p* < 0.001; R2, coefficient of determination = 0.845, *p* < 0.001.

Significant moderate to strong correlations were observed between physical activity and almost all the variables assessed from the body composition analysis (Table 2). Briefly, phase angle showed the largest magnitude of correlation with sedentary time (negative and moderate, *r* = –0.559; *p* = 0.038) and total time spent in MVPA (positive and strong, *r* = 0.919; *p* < 0.001) (Figure 3).

**TABLE 2:** Pearson’s product-moment correlation coefficient (r) between physical activity domains and selected parameters of body composition analysis.

	Sedentary time	BMI	OH	LTI	FTI	PH
MVPA	−0.459	0.709**	−0.743**	**0.738** **	0.406	0.919**
BMI	−0.051		–0.531	0.413	**0.858** **	0.666**
OH	0.413			–0.366	–0.468	−0.745**
LTI	−0.573*				–0.101	0.812**
FTI	0.203					0.323
PH	−0.559*					1

MVPA, moderate- to vigorous physical activity; BMI, body mass index; LTI, lean tissue index; FTI, fat tissue index; PH, phase angle; OH, overhydration; *p value < 0.05; **p value < 0.001.

Furthermore, among the selected serum parameter variables, none correlated with sedentary time, while only IL6 showed a moderate negative correlation with total time spent in MVPA (*r* = –0.576; *p* = 0.031). Finally, measures of physical fitness showed no significant correlation with sedentary time or time spent in MVPA.

## Discussion

This pilot study examined the association among objectively measured physical activity, selected measures of physical fitness, body composition, and disease burden in HD patients; moreover, it also examined the magnitude of differences in physical activity between dialysis and non-dialysis days and between subjective characteristics of the 14 HD patients reaching and not reaching physical activity. We found no significant differences between males and females in all the observed variables with the exception of muscle strength (handgrip). Very interesting is the finding between HD patients reaching and those not reaching guidelines where the patients who did not reach the guidelines showed reduced lean body mass and hyperhydration; MVPA decline is very high on dialysis days compared to non-dialysis days (–49%); no significant correlations were found between serum parameters, symptom, and comorbidity burden, but a very large and significant positive correlation was found between phase angle and total MVPA, thus reinforcing a potential useful role of monitoring phase angle in dialysis patients.

Hemodialysis patients in present study engaged in an average of 35.3 min/day of moderate to vigorous physical activity (MVPA), while referenced healthy adults, also measured by the Actigraph triaxial accelerometer and using the same threshold as in the present study (Chomistek et al., 2017), engaged in an average MVPA of 41.6 min/day. It has long been known that HD patients are debilitated (Gutman et al., 1981) and report low levels of physical activity, assessed with questionnaires (DeOreo, 1997), resulting in atrophy and reduced functioning (Diesel et al., 1993; Moore et al., 1993). Newer studies that have used accelerometers to measure physical activity in HD patients have shown inconsistent results due to differences in the devices used, poorly reported valid wearing times, and varying minimum valid days for measurement (Young et al., 2019). Therefore, comparison with previous studies regarding average MVPA in HD patients was difficult. Generally, our results are consistent with the findings of Kim et al. (2014), who reported significantly lower daily MVPA for almost 27% on dialysis days with no significant differences between genders. Our data, however, show a much larger reduction in MVPA on dialysis days (22 percentage point difference, a relative change for a factor of 1.81). This is probably due to coronavirus disease-2019 (COVID-19) lockdown and relatively high physical activity levels on non-dialysis days in this sample. Physical inactivity on dialysis days can lead to detrimental musculoskeletal and cardiac effects and reduced physical work capacity; it can exacerbate skeletal muscle wasting, cardiac dysfunction, loss of bone mass, and glucose intolerance (Krasnoff and Painter, 1999). Given the strong evidence regarding the association of lower levels of physical activity with higher mortality risk in HD patients (O’Hare et al., 2003), this large reduction in physical activity on dialysis days may be of significant importance. We feel that this perspective is a key argument to widely implement intra-dialysis exercise either immediately prior to or during dialysis (Bogataj et al., 2020) to reduce the deficit in physical activity on dialysis days. Moreover, raising physical activity levels in HD patients could also be beneficial since, a satisfactory amount of physical activity reduces the risk of cardiovascular disease in general population (Kraus et al., 2002).

Phase angle (a parameter that is sensitive to both reduction in lean body mass and increase in overhydration) showed the strongest correlation with total time spent on MVPA, which is one of the most important measures assessed by accelerometry. In accordance with this, we found 27% smaller phase angle together with increased overhydration and reduced lean mass in patients not achieving the physical activity recommendations. Phase angle is an independent parameter calculated from the resistance and reactance of bioimpedance measurements and is postulated to reflect overall cellular health, with higher values indicating better cell function and cell wall integrity (De Lorenzo et al., 1997; Norman et al., 2012). It has been suggested to be a marker of muscle quality, health, and functionality in both cross-sectional and longitudinal studies (Nunes et al., 2019; Tomeleri et al., 2019). In dialysis population, lower levels of phase angle predicted impaired muscle function, reduced health-related quality of life, impending hospitalization, and mortality (Beberashvili et al., 2014). Although the main focus of this study was to objectively examine physical activity in HD patients and possible associations with some selected measures of body composition, it should be noted that there is a proven causal relationship between physical activity and phase angle (Mundstock et al., 2019). This study extends this finding and shows a very large positive association between phase angle and physical activity in dialysis patients. Even though it has appeared in some previous publications that physical activity causally improves phase angle values through different mechanisms (Mundstock et al., 2019), it should be noted that the reverse causality (patients with healthier body composition and larger phase angles can engage at higher levels of habitual physical activity) may be also in effect. Evidently, with our study having an observational design, we are not able to provide any conclusion on the causality of this association, and the association shown in Figure 3 should be interpreted within this limitation. It is, however, most probable that both directions of causality operate here, and that this would increase the importance of regular follow-up of phase angle in the dialysis population.

### Strengths and Limitations

Strengths of this study included using precise physical activity measurement equipment, rigorous inclusion protocol, consistent measurement criteria, and body composition control. However, some limitations have been acknowledged: (i) patients were aware that they were being measured and monitored, thus their physical activity patterns could be increased above their regular habitual level; (ii) high measurement and equipment costs limited the availability of ActiGraph sensors and did not allow for the measurement of physical activity of all included patients during the same week, which means that the patterns of physical activity could be affected by different weather conditions; (iii) external factors, such as family problems, friendship problems, dietary habits and changes in environment, and socioeconomic status, could influence physical activity; (iv) during the measurements, the country was subjected to some COVID-19 related restraints; (v) the size of the sample is small, so we cannot freely generalize the results to the population of HD patients. Moreover, for the smallest correlation obtained in this pilot study (∼0.5), 26 patients are required to demonstrate the relationship independent of gender with a probability of alpha error ≤ 0.05 and a power of test ≥ 0.8; (vi) only HD patients were included in the study, so we cannot generalize the results to peritoneal dialysis population; (vii) however, we believe that this pilot effort was worthwhile, since the results do support the need to conduct a larger study, primarily to confirm whether there really are also such large differences between dialysis and non-dialysis days in other social and ethnic HD and peritoneal dialysis groups, with the aim of consolidating the need for systematic introduction of pre- and inter-dialysis exercises.

## Conclusion

In this study, we used objective measure of physical activity with consistent measurement criteria in the pilot sample of HD patients and found a large decrement in the time spent in moderate and vigorous physical activity on dialysis days. This should support the need for ubiquitous uptake of pre- or intra-dialysis exercise on dialysis days in HD units. Patients with low lean tissue mass and increased extracellular overhydration engaged in lowest levels of physical activity and it is patients with these body composition characteristics who should be first to receive attention by caregivers, renal care staff, and kinesiologists to be included in structured physical exercise programs together with dietary intervention to cover for increased energy expenditure. While we could not show an association of physical activity levels with measures of symptom burden or relevant biochemical parameters in this sample size, phase angle convincingly and, to a high extent, correlated best with the time spent in moderate-vigorous activity and should be studied further as possibly one of the most useful markers of habitual physical activity of dialysis patients.

## Data Availability Statement

The raw data supporting the conclusions of this article will be made available by the authors, without undue reservation.

## Ethics Statement

The studies involving human participants were reviewed and approved by Slovenian National Medical Ethics Committee (ref. no. 0120-430/2019/12). The patients/participants provided their written informed consent to participate in this study.

## Author Contributions

VS and ŠB conceptualized the design of the study and recruited subjects into the study. JP conducted the research. AP analyzed and interpreted the data and drafted the manuscript. VS, ŠB, AP, and JP drafted and reviewed the manuscript. JR analyzed the data and conducted the research. MP interpreted the data and supervised the research. All authors have read and approved the final version of the manuscript.

## Conflict of Interest

The authors declare that the research was conducted in the absence of any commercial or financial relationships that could be construed as a potential conflict of interest.

## Publisher’s Note

All claims expressed in this article are solely those of the authors and do not necessarily represent those of their affiliated organizations, or those of the publisher, the editors and the reviewers. Any product that may be evaluated in this article, or claim that may be made by its manufacturer, is not guaranteed or endorsed by the publisher.
